# Evaluation of Potential Genotoxicity of HIV Entry Inhibitors Derived from Natural Sources

**DOI:** 10.1371/journal.pone.0093108

**Published:** 2014-03-25

**Authors:** Elena E. Paskaleva, Manoj Arra, Yanze Liu, Huijun Guo, Glenn Swartz, Jeffrey S. Kennedy, Curt Breneman, Alexander Shekhtman, Mario Canki

**Affiliations:** 1 Palm Biologicals, LLC, Albany, New York, United States of America; 2 Center for Biotechnology & Interdisciplinary Studies, Rensselaer Polytechnic Institute, Troy, New York, United States of America; 3 Center for Immunology and Microbial Disease, Albany Medical College, Albany, New York, United States of America; 4 Institute of Medicinal Plant Development, Chinese Academy of Medical Sciences & Peking Union Medical College, Beijing, China; 5 First Affiliated Hospital of Henan University of Traditional Chinese Medicine, Zhengzhou, China; 6 Advanced Bioscience Laboratories, Inc., Rockville, Maryland, United States of America; 7 Translation Medicine, Albany Medical College, Albany, New York, United States of America; 8 Department of Chemistry, Rensselaer Polytechnic Institute, Troy, New York, United States of America; 9 Department of Chemistry, State University of New York at Albany, Albany, New York United States of America; Chinese Academy of Medical Sciences, China

## Abstract

AIDS is a global pandemic that has seen the development of novel and effective treatments to improve the quality of life of those infected and reduction of spread of the disease. Palmitic Acid (PA), which we identified and isolated from *Sargassum fusiforme,* is a naturally occurring fatty acid that specifically inhibits HIV entry by binding to a novel pocket on the CD4 receptor. We also identified a structural analogue, 2-bromopalmitate (2-BP), as a more effective HIV entry inhibitor with a 20-fold increase in efficacy. We have used the structure-activity relationship (SAR) of 2-BP as a platform to identify new small chemical molecules that fit into the various identified active sites in an effort to identify more potent CD4 entry inhibitors. To validate further drug development, we tested the PA and 2-BP scaffold molecules for genotoxic potential. The FDA and International Conference on Harmonisation (ICH) recommends using a standardized 3-test battery for testing compound genotoxicity consisting of the bacterial reverse mutation assay, mouse lymphoma assay, and rat micronucleus assay. PA and 2-BP and their metabolites tested negative in all three genotoxicty tests. 2-BP is the first derivative of PA to undergo pre-clinical screening, which will enable us to now test multiple simultaneous small chemical structures based on activity in scaffold modeling across the dimension of pre-clinical testing to enable transition to human testing.

## Introduction

After investigating a large number of natural products, we showed *Sargassum fusiforme* (*S. fusiforme*) algae to have HIV inhibiting properties [1], [2]. From *S. fusiforme* we isolated and identified palmitic acid (PA) as a bioactive molecule that inhibited both X4- and R5-tropic HIV entry to CD4^+^ cells [3]. We also identified 2-bromopalmitate (2-BP) as a more potent analog, which showed approximately 20-fold increase in efficacy as compared to PA [4]. We demonstrated specificity of PA-to-CD4 receptor binding by NMR saturation transfer spectroscopy (STD-NMR) [3], and the inhibition of HIV entry by CD4-to-gp120 competition ELISA experiments [5]. Utilizing *in silico* molecular modeling involving a combination of binding assay data, alpha site analysis and docking/scoring approaches, we have identified a novel druggable cavity on the CD4 receptor that binds both PA and 2-BP and inhibits gp120-to-CD4 binding, thus blocking HIV entry [4]. Considering that the CD4 glycoprotein is the obligatory HIV receptor regardless of HIV coreceptor usage, the PA and 2-BP represent small chemical entity (SCE) molecule binding to the specified CD4 cavity that explains the observed entry inhibition of both X4- and R5-tropic HIV [3], [4]. We also showed inhibition of R5 HIV-1 productive infection in human cervix tissue *ex vivo* model experiments, demonstrating opportunity for topical microbicide development aimed at preventing sexual HIV transmission [5], which remains the main cause of HIV transmission.

Preliminary data with 2-BP indicates that the identified multiple CD4 pockets capable of hosting inhibitors with nanomolar potencies [4], allowing us to use these sites to test various optimized ligands within a platform in order to develop safe and potent CD4 entry inhibitors. Taken together, PA and 2-BP represent useful scaffold molecules in defining structure-activity relationship to identify new more potent small chemical entities for further development and optimization that would mitigate HIV transmission at the point of contact.

Palmitic acid is not unique to *S. Fusiforme*, and is found across nature as one of the most common saturated fatty acids. It is present in low concentrations (≈1.88 nM), in human plasma, with most of it bound to albumin [6]. By itself, palmitic acid has been studied for a variety of effects, both positive and negative. It has been linked to potential anti-inflammatory properties by inhibiting phospholipase A (PLA) enzyme [7], however it was also associated with the release of pro-inflammatory signals in cell culture [8]. It has been shown to induce apoptosis in hepatocytes, nerve cells and myofibrils [9]–[12]. Clinically, PA has anti-microbial properties [13] and has been linked to the development of cardiovascular disease by elevating plasma cholesterol and low-density lipoprotein (LDL) levels [14]. One study showed that palm oil, which contains approximately 44% palmitate, led to increased coronary heart disease and tumor growth [15], indicating that saturated fatty acids when consumed at high levels can lead to disease in humans. These studies highlight the need for greater understanding of PA and 2-BP like SCE scaffold molecules, which may exert additional activity on the CD4 molecule, providing a binding and blocking site for HIV entry without effecting CD4 functional activity.

The ICH and FDA approved guidelines for testing of compounds using a 3-test battery including a bacterial reverse mutation test, an *in vitro* mammalian mouse lymphoma gene mutation assay (MLA), and an *in vivo* rat micronucleus assay. The ICH guidelines also recommend parameters and criteria for determining genotoxicity of compounds and their metabolites using metabolic activation by Aroclar-induced Rat Liver S9 Fraction. The bacterial reverse mutation assay or Ames test is used to determine point and frameshift bacterial mutations [16], [17]. *In vitro* MLA, [18] uses mouse lymphoma cells with a heterozygous thymidine kinase (tk) locus on chromosome 11 [19], and tests for the number of mutants with an inactivated tk^+^ allele. The assay can detect various types mutations in chromosome 11 including frameshift and base pair substitutions, deletions and translocations [19]. The *in vivo* rat micronucleus assay measures the number of micronuclei present in polychromatic erythrocytes (PCE) from rat bone marrow [20], [21], and is used in determining potential carcinogenicity of compounds and their ability to cause chromosomal damage in replicating cells [22]–[24].

In the present study we tested the mutagenic potential of PA and 2-BP scaffold models using the bacterial reverse mutation assay, MLA, and Rat Micronucleus test, in order to validate their potential for further drug development.

## Materials and Methods

### Ethics Statement

This study is not duplicative or unnecessary. The number of rats as well as the procedures and experimental design used for this study have been reviewed and were approved by the BioReliance Institutional Animal Care and Use Committee (Protocol # 8 and # 10). All procedures involving animals performed at BioReliance follow the specifications recommended in The Guide for the Care and Use of Laboratory Animals (National Academy Press, Washington, D.C., 1996) 4.

Animals were housed in an AAALAC accredited facility with a controlled environment of 50±20% relative humidity and 72±3°F with a 12 hour light/dark cycle. The animal rooms were supplied with at least 10 changes of fresh HEPA-filtered air every hour.

Rats of the same sex were housed up to two per rodent Micro-Barrier cage. Cages were placed on the racks equipped with an automatic watering system and Micro-VENT full ventilation, HEPA filtered system. The purpose of this system was to supply uninterrupted positive air to each individual rodent Micro-Barrier cage and to capture the effluent air from each cage and re-filter the air (HEPA) prior to introducing the air back into the cage/room.

Heat treated Sani-Chip hardwood chips were used for bedding to absorb liquids (P.J. Murphy Forest Products, Montville, NJ). Bedding was analyzed by the Manufacturer for any contaminants.

Animals were allowed free access to tap water, which meets U.S. EPA drinking water standards [water source is Washington Suburban Sanitary Commission (WSSC) Potomac Plant]. Drinking water was monitored at least annually for levels of specified microorganisms, pesticides, heavy metals, alkalinity and halogens.

A certified laboratory rodent chow (Harlan 2018C Certified Global Rodent Diet) was provided ad libitum. The food was analyzed by the manufacturer for the concentrations of specified heavy metals, aflatoxin, chlorinated hydrocarbons, organophosphates and specified nutrients.

The results of bedding, food and water analyses are on file at BioReliance. There are no contaminants to the bedding, feed and water that are expected to interfere with the study.

### Chemicals and Solubility

PA and 2-BP were obtained from Sigma, and dimethyl sulfoxide (DMSO) was selected as the solvent of choice based on previous cell culture viability and compatibility with target cells. Both test articles formed a clear and soluble solution in DMSO from 0.0003 to 100 mg/mL.

### Bacterial Reverse Mutation Assay

The tester strains used in this assay were the *Salmonella typhimurium* histidine auxotrophs TA98, TA100, TA1535 and TA1537, and *Escherichia coli* WP2 *uvr*A. Between 1.5×10^8^ and 2×10^8^ cells were plated per system. Each test system was exposed to test article and controls in duplicate via the plate incorporation methodology as previously described [16], [17]. Plates were inverted for 48–72 hours before the number of reverse mutants was measured by counting. The maximum dose tested was 5000 μg/plate, which was achieved using a concentration of 100 mg/mL and 50μl plating aliquot with overnight cultures of each tester strains on selective minimal agar in the presence or absence of Aroclor-induced rat liver S9.

The condition of the bacterial background lawn was evaluated for evidence of test article toxicity by using a dissecting microscope. Revertant colonies for a given tester strain and activation condition were counted either entirely by automated colony counter with 0.08 mm colony size detection sensitivity or entirely by hand unless the plate exhibited toxicity. Plates with sufficient test article precipitate to interfere with automated colony counting were counted manually. All historical criteria for a valid test were met.

### L5178Y/TK^+/−^ Mouse Lymphoma Assay

L5178Y/TK^+/−^ mouse lymphoma cells ATCC clone 3.7.2C was prepared for the assay as previously described [18], [19], [25]–[27]. The mutagenic potential of the test articles (PA and 2-BP) was measured by its ability to induce TK^+/−^ → TK^−/−^ (trifluorothymidine (TFT)-resistant phenotype) mutations. For those test articles demonstrating a positive response, mutant colonies were sized as an indication of mechanism of action. The mammalian mutation assay was performed by exposing single cultures of L5178Y/TK^+/−^ cells to eight different concentrations of each test article as well as positive and negative (solvent) controls. Exposures were for 4 hours in the presence of a metabolic (S9) activation system and 24 hours in the absence of S9 activation. Following a two-day expression period, with daily cell population adjustments, cultures demonstrating 0% to 90% growth inhibition were cloned, in triplicate, in both complete medium (Fischer’s Medium for Leukemic Cells of Mice with 0.1% Pluronics, supplemented with 10% horse serum, 2 mM L-glutamine, 100 U penicillin/mL and 100 μg streptomycin/mL (F0P)) and selective medium (F0P with 3μg/mL trifluorothymidine) containing 0.22–0.23% soft agar. For selection of the TK^−/−^, cells from at least 6 cultures were plated into dishes at a density of 1×10^6^ cells/100mm plate in cloning medium containing 0.22% to 0.23% agar and 2–4 μg TFT/mL. For estimation of cloning efficiency at the time of selection, 200 cells/100mm plate were plated in triplicate in cloning medium free of TFT (viable cell (VC) plate). Plates were incubated for 10–14 days, and after selection period the colonies were enumerated using an automated counter with 0.08 mm colony size detection sensitivity. The diameters of the TFT colonies from the positive control and solvent control cultures were determined over a range of approximately 0.2 to 1.1 mm. Induced mutant frequency for the test articles was calculated by subtracting mean solvent mutant frequency from the total mutant frequency for the test articles.

### Rat Bone Marrow Erythrocyte Micronucleus Test

The test articles PA and 2-BP, were evaluated for genotoxic potential as measured by its ability to increase the incidence of micronucleated polychromatic erythrocytes (MPCEs) in bone marrow of male and female Sprague-Dawley (NTac:SD) rats. The micronucleus test was composed of two phases: a confirmatory toxicity phase and a definitive phase (micronucleus study). This study was conducted in compliance with the testing guidelines (International Conference on Harmonisation 2012). The definitive micronucleus study was conducted using established and validated procedures [20]–[22], [28]. The purpose of the confirmatory study was to evaluate the toxicity of the test articles at a dose of 2000 mg/kg that is the highest dose recommended by guidelines (OECD Guideline 474, 1998). The test article vehicle, 1% Carboxymethylcellulose/0.1% Tween 80 in deionized water, was selected based on good workability of the test article in the vehicle and compatibility with the test system and route of administration. In the definitive micronucleus study, the vehicle was also used as vehicle control and cyclophosphamide, 40 mg/kg (a known clastogen, used in over 99% of the assays) was used positive control article. In both phases of the study, the test or control articles were administered at a constant volume of 20 ml/kg body weight, by a single oral feeding. Rats were observed after dose administration and during the course of the study for clinical signs of toxicity.

For PA, based on the available LD50 data, rats tolerated doses >2000 mg/kg (LD50 in rats >10,000 mg/kg, as per MSDS). In order to confirm this information, 5 male and 5 female rats were exposed orally to PA at a dose of 2000 mg/kg, and no mortality was observed confirming MSDS data. For PA the definitive study consisted of 8 groups, each composed of 5 male and 5 female rats. Animals were exposed to the vehicle or test article at 500, 1000 and 2000 mg/kg, male and female rats in control group were exposed to Cyclophosphamide at 40 mg/kg and were euthanized at 24 hour post-dose. Five animals/sex were dosed with the vehicle or the test article at 2000 mg/kg were euthanized at 48 hour post-dose. No mortality was observed in either group prior to 24 or 48 hour scheduled time point.

For 2-BP test article in the confirmatory study, 5 male and 5 female rats were orally exposed to 2-BP at a dose of 2000 mg/kg. Due to mortality at a dose of 2000 mg/kg in the confirmatory study, a dose of 1800 mg/kg was tested as the highest dose in the definitive micronucleus study. Animals were exposed to the vehicle or the test article at 450, 900 and 1800 mg/kg, and were euthanized at 24 hour post-dose. Five animals were dosed with the vehicle or the test article at 1800 mg/kg, were euthanized at 48 hour post-dose.

For both test articles, at the scheduled euthanasia time point, femoral bone marrow was collected; bone marrow smears (slides) were prepared and stained with Acridine orange stain (a nucleic acid specific stain). Bone marrow cells [polychromatic erythrocytes (2000 PCEs/animal)] were examined microscopically for the presence of micronuclei (micronucleated PCEs; MPCEs). A statistical analysis of data was performed using the binomial distribution (Kastenbaum-Bowman Tables, p≤0.05) [29].

## Results

### Bacterial Reverse Mutation Assay

The assay was performed as originally described by Ames et al. (1975) and updated by Maron and Ames (1983)[16], [17]. We utilized this assay to determine frameshift and point mutations in bacteria caused by increasing concentrations of PA and 2-BP, from 0 to 5000 μg/plate, with and without metabolic activation using Rat Liver S9 (Table 1). The tester strains used were *S. Typhimurium* strains TA98, TA100, TA1535, TA1537, and *E. Coli* strain WP2 uvrA. All strains used are auxotrophic mutants that require histidine for growth, and after treatment only mutants that gain the ability to produce histidine are selected as reverse mutants. As per accepted ICH guidelines, a compound is considered mutagenic if the mean number of revertants is three times greater than vehicle control value for strains TA1535 and TA1537 and two times greater than vehicle control value for strains TA98, TA100 and WP2, with or without metabolic activation. The formation of precipitate above 500 μg/plate without metabolic activation and at 5000 μg/plate with S9 activation was evaluated by visual examination without magnification, though it was not considered to adversely impact the results. During the assay, no toxicity and no contaminant colonies were observed. Treatment with concentrations of PA and 2-BP up to 5000 μg/plate did not reach a significant increase in reverse mutants over the vehicle control group. Based on these results these compounds are considered to be not mutagenic.

**Table 1:** Bacterial Reverse Mutation Assay with and without metabolic activation.

A.	PA without activation						C.	2-BP without activation					
	Dose(μg/plate)	TA98	TA100	TA1535	TA1537	WP2		Dose(μg/plate)	TA98	TA100	TA1535	TA1537	WP2
	**Vehicle**	14±2	222±29	13±4	7±7	34±4		**Vehicle**	11±5	172±11	7±1	9±4	24±6
	**Positive**	124±10	673±47	700±22	447±3	617±12		**Positive**	54±4	519±19	384±3	206±33	349±35
	0.015	14±2	200±12	11±2	11±1	44±13		0.015	8±1	177±4	16±4	3±1	33±4
	0.05	22±3	235±0	8±4	6±2	48±10		0.05	10±6	190±11	17±8	2±0	42±6
	0.15	12±6	179±15	10±1	5±1	44±6		0.15	7±7	177±12	17±1	5±0	24±1
	0.5	14±0	220±6	10±1	8±4	44±6		0.5	13±6	163±11	12±7	3±0	33±0
	1.5	16±2	231±14	6±1	8±2	43±3		1.5	10±1	172±24	15±4	3±0	27±4
	5	14±0	192±18	6±1	6±5	57±1		5	14±4	133±28	18±8	3±1	21±13
	15	12±3	205±23	8±9	8±1	50±7		15	7±2	129±35	11±3	4±1	28±4
	50	22±1	187±13	4±4	5±1	49±5		50	5±1	67±6	11±2	4±4	36±20
	150	13±4	179±1	10±4	10±6	37±4		150	5±0	26±8	15±4	14±13	24±13
	500	14±2	187±5	9±0	4±0	44±11		500	4±0	19±12	8±0	12±1	36±3
	1500	15±0	205±1	10±0	10±1	56±6		1500	6±1	10±1	8±1	6±1	27±1
	5000	13±3	125±13	12±4	7±1	40±1		5000	6±1	8±1	7±0	5±1	24±7
**B.**	**PA with S9 activation**						**D.**	**2-BP with S9 activation**					
	**Dose** **(μg/plate)**	**TA98**	**TA100**	**TA1535**	**TA1537**	**WP2**		**Dose** **(μg/plate)**	**TA98**	**TA100**	**TA1535**	**TA1537**	**WP2**
	**Vehicle**	18±8	204±13	8±1	7±1	35±6		**Vehicle**	24±6	184±1	8±1	4±2	39±18
	**Positive**	248±20	830±76	60±14	40±4	109±13		**Positive**	155±11	712±66	63±9	23±1	131±12
	0.015	19±2	192±2	14±2	3±1	49±1		0.015	25±11	198±3	9±4	6±0	35±4
	0.05	23±2	194±18	4±2	7±3	56±8		0.05	25±1	196±16	7±4	7±0	42±4
	0.15	25±8	199±6	11±5	6±2	49±18		0.15	21±2	176±16	11±4	6±4	36±1
	0.5	24±9	193±5	8±1	4±4	60±11		0.5	20±10	161±7	10±4	4±2	33±4
	1.5	21±8	219±23	10±0	6±2	49±2		1.5	18±7	191±2	10±6	9±1	35±1
	5	21±0	216±16	11±0	8±2	47±4		5	17±1	155±13	9±0	8±1	30±1
	15	21±1	184±30	13±4	9±3	50±4		15	17±1	175±6	13±1	5±4	32±2
	50	19±3	165±6	8±1	7±3	54±1		50	15±4	151±6	10±2	5±2	49±6
	150	11±1	216±49	10±6	7±0	57**±**12		150	13±4	104±5	10±6	9±4	31±1
	500	15±6	225±6	12±3	4±2	43±3		500	8±1	45±4	11±4	6±3	37±1
	1500	14±4	218±12	15±0	6±5	57±1		1500	12±0	22±5	15±4	9±1	37±8
	5000	13±5	254±18	6±0	5±1	28±3		5000	9±1	15±8	13±1	5±1	27±4

*S. Typhimurium* strains TA98, TA100, TA1535, TA1537 and *E. Coli* strain WP2 were incubated with increasing concentrations of test article with or without activation, as indicated. PA and 2-BP were dissolved in DMSO at 100 mg/ml and added to each plate at the indicated concentration for overnight incubation. Reverse mutations were selected and quantified on histidine negative plates. Vehicle negative control and positive control are indicated at the top of the table. Representative of two separate experiments, all data are mean +/− SD. The positive control for strain TA98 with and without metabolic activation was 2-nitrofluorene (1 μg/plate) and 2-aminoanthracene (1 μg/plate), respectively. The positive control for strain TA100 with and without metabolic activation was sodium azide (1 μg/plate) and 2-aminoanthracene (1 μg/plate), respectively. The positive control for strain TA1535 with and without metabolic activation was sodium azide (1 μg/plate) and 2-aminoanthracene (1 μg/plate), respectively. The positive control for strain TA1537 with and without metabolic activation was 9-aminoacridine (75 μg/plate) and 2-aminoanthracene (1 μg/plate), respectively. The positive control for strain WP2 with and without metabolic activation was methyl methanesulfonate (1000 μg/plate) and 2-aminoanthracene (10 μg/plate), respectively.

### Mouse Lymphoma Assay

**Figure 1:**
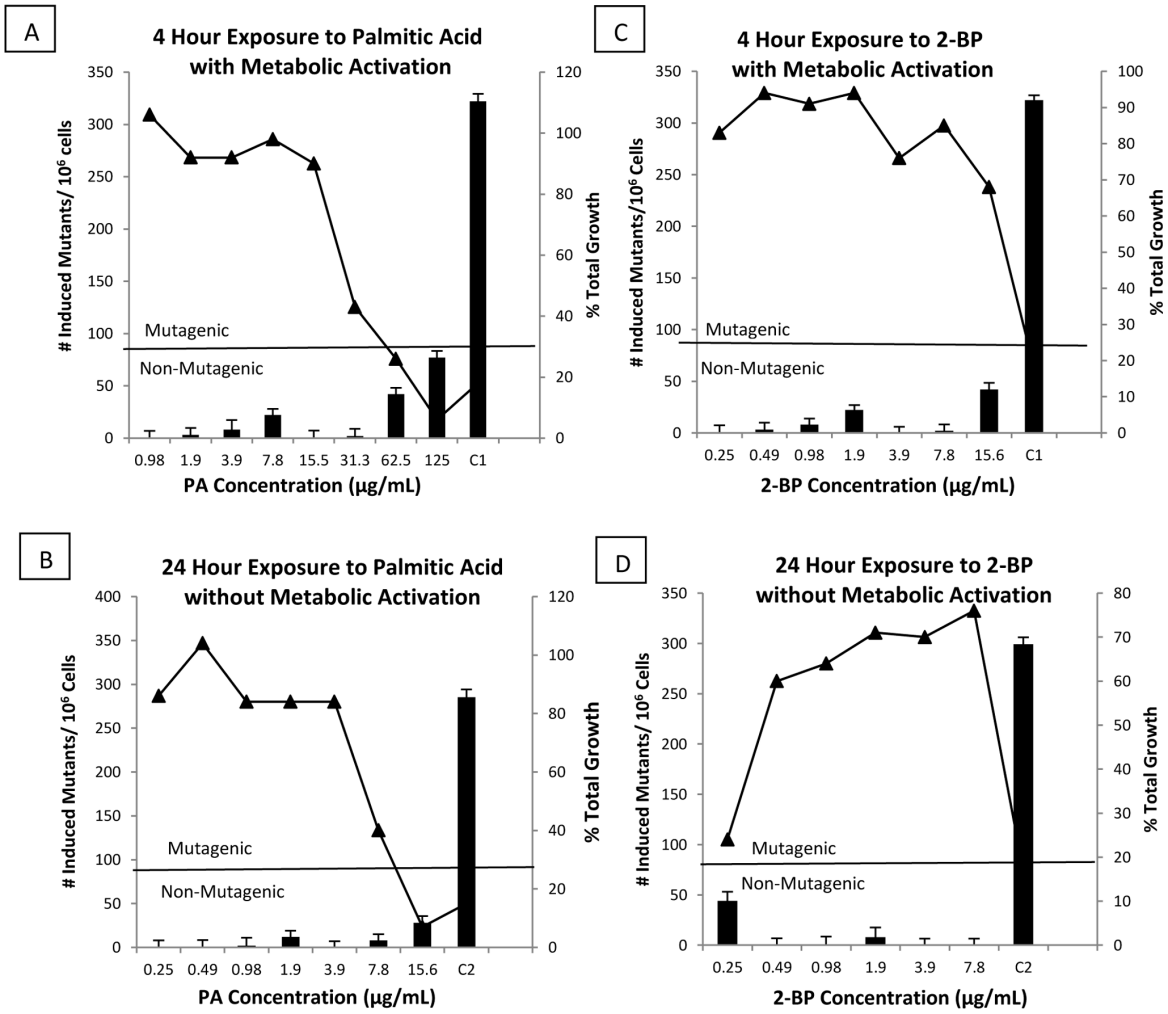
*In Vitro* Mouse Lymphoma Assay. Both PA and 2-BP were tested for mutations in a 4 hour exposure with metabolic activation (A and C) or for 24 hour exposure without metabolic activation (B and D). Number of induced mutants/10^6^ cells are displayed on the left hand y-axis and indicated by bars. Values above 90 mutants/10^6^ cells (Indicated by horizontal line) were considered to be mutagenic. Positive control for activation (C1 = 1 μg/mL DMBA), and without activation (C2 = 5 μg/mL MMS). Representative of three experiments, all data are mean +/− SD. The right hand axis displays cell toxicity as a function of total growth, represented by (-▴-) line. Concentrations with less than 10% total growth were considered cytotoxic and were not used to determine mutagenicity.

The MLA was performed to test PA and 2-BP for point mutations and alterations in chromosomal structure in the thymidine kinase locus (Figure 1). In order to satisfy the criteria for a mutagenic response, cultures with at least 10% total growth must display concentration dependent increase in mutations that are greater than 90 mutants per 10^6^ cells [26]. Cloning efficiency for all experiments was greater than 80% (not shown), and mutant frequencies for vehicle and positive control were not significantly different from historical values [21], [23], [24]. Positive controls Methyl Methanesulfonate (MMS) and 7,12-dimethylbenz(a)anthracene (DMBA) yielded the expected increase in small and large colonies. Total growth of mouse lymphoma cells must be greater than 10% to determine mutagenicity at a given concentration (Right Y-axis, total growth represented by a solid line). Less than 10% total growth existed only at 125 μg/mL and 15.6 μg/mL for PA with and without S9 activation, however neither displayed mutagenicity, respectively (Figure 1, A and B). At all other tested concentrations for both compounds, with and without metabolic activation, growth was greater than 10%, indicating that neither compound inhibited cell growth. Test cultures for both compounds exhibited no significant mutant frequencies over vehicle control in both activated and non-activated system, (Figure 1, left Y-axis represented by bars). Based on these results we conclude that PA and 2-BP are not mutagenic in the MLA.

### Rat Micronucleus Analysis

**Figure 2:**
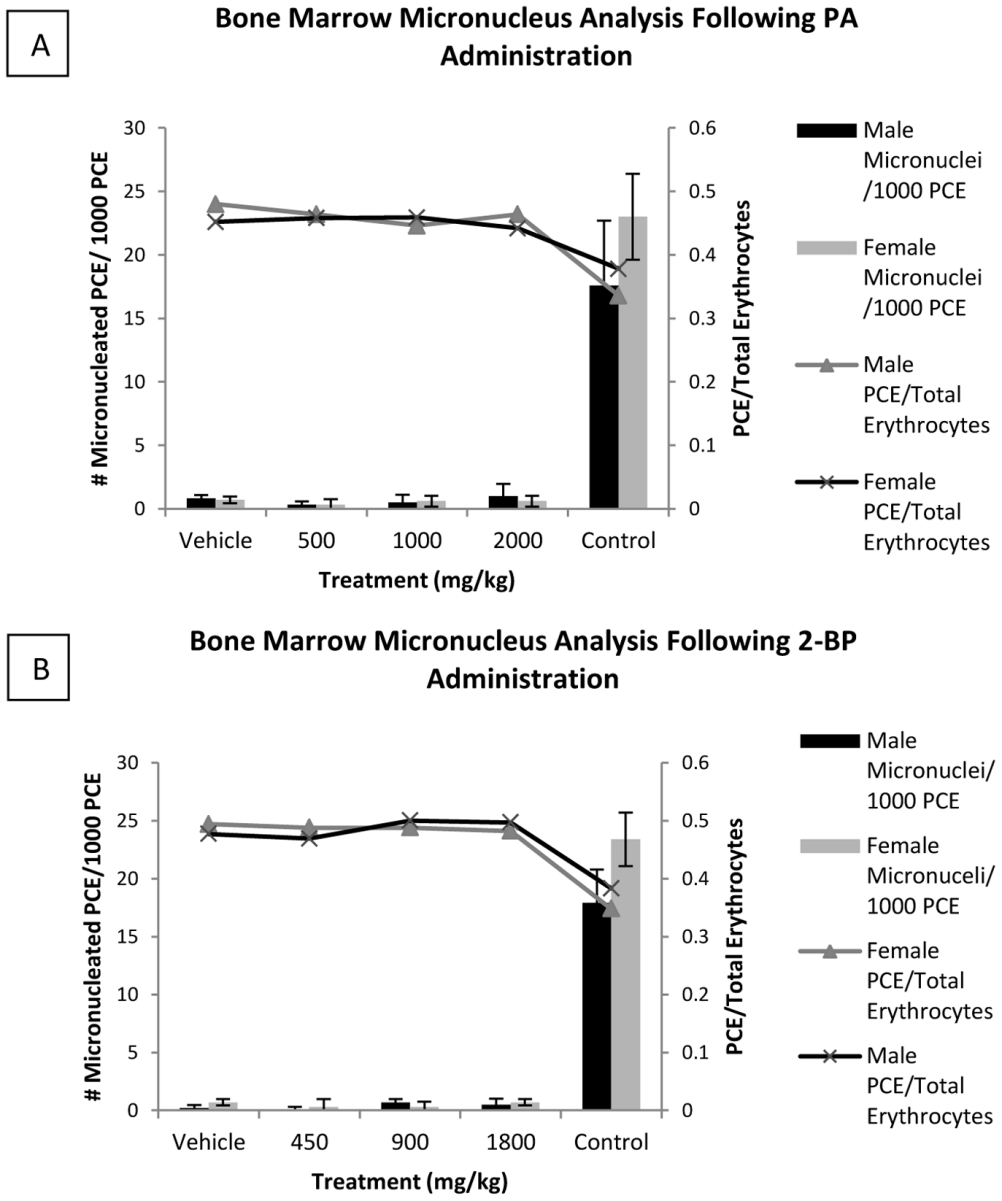
Mammalian *In Vivo* Rat Micronucleus Assay. Rat PCE/total erythrocyte (right hand y-axis) after 24 hours exposure to increasing concentrations of PA (A) or 2-BP (B), for male (-▴-), and female (-x-) rats. The number of MPCEs/1000 PCEs (left hand y-axis) were counted in rats of each sex and represented by bars. Representative of three experiments, all data are mean +/− SD. Vehicle was used as a negative control and cyclophosphamide was used as a positive control with male and female rats reported seperately.

Next we wanted to evaluate the clastogenic/aneugenic impact of the test articles as measured by their ability to induce micronucleated polychromatic erythrocytes (MCPEs) in rat bone marrow (Figure 2). In the confirmatory study the maximum dose for PA and 2-BP was determined to be 2000 mg/kg and 1800 mg/kg, respectively. Rats were orally fed increasing concentrations of PA and 2-BP, and 24 hours after feeding the ratio of polychromatic erythrocytes (PCEs) to total erythrocytes was measured to determine the effect on normal erythropoiesis (right hand y-axis). The bone marrow analysis showed no appreciable reductions in the ratio of PCE’s to total erythrocytes in the PA and 2-BP treatment groups relative to respective vehicle controls, indicating that both test articles do not significantly inhibit erythropoiesis. Next, PCEs were examined microscopically for presence of micronuclei. There was no statistically significant (p>0.05, Kastenbaum-Bowman Tables) increase in the incidence of MPCE’s in the PA and 2-BP groups (left hand y-axis bars). Rats were also tested at 2000 mg/kg and 1800 mg/kg PA and 2-BP, respectively after 48 hours and showed no increase in MCPE’s (not shown). Cyclophosphamide (positive control) induced a statistically significant decrease in erythropoiesis and increase in the incidence of micronucleated PCEs (p≤0.05, Kastenbaum-Bowman Tables) in both male and female rats. The number of MPCEs in the vehicle control groups did not exceed historical values, validating test as specified by the ICH guidelines. Based on these results, we conclude that PA and 2-BP are not mutagenic in either male or female rats.

## Discussion

HIV entry inhibitors are desirable therapeutic approaches that block virus from entering target cells and thereby prevent *de novo* infections and also limit both viral integration and subsequent viral spread in the already infected host. Screening large libraries of compounds to find potential SCE CD4 inhibitors is burdened by the potential for these high throughput screens to identify SCEs that will also interfere with CD4 function. Our *in silico* platform is unique by identifying a model structure with active sites that potentially do not interfere with CD4 function, considering that the pharmacophore of a molecule has necessary core features such as structure or shape required for the desired activity against a target [30]. Once model molecule is identified and the structure-activity relationship is understood, *in silico* modeling, *de novo* design and synthesis, and *in vitro* testing can be rapidly performed to achieve the desired therapeutic index (TI) and pharmacokinetic profile. This type of scaffold-based approach is used extensively in basic research to identify more potent compounds [31], [32].

In previous reports we identified PA as a natural SCE that specifically binds to the CD4 receptor cavity and inhibits HIV entry by blocking CD4-to-gp120 binding [3]. Utilizing PA as scaffold model molecule to improve micromolar inhibition efficacy, we identified 2-BP as an analog with nanomolar efficacy of binding CD4 and blocking virus entry [4]. Testing fatty acids that were shorter or longer than 16 carbons did not inhibit HIV entry, indicating that the specific scaffold length of PA and 2-BP was necessary for proper fit to the identified CD4 receptor cavity. These results indicate that the CD4 site is druggable and capable of hosting specific inhibitors with nanomolar potencies, allowing us to use these sites (and the structures of tested ligands) to develop leads that could mitigate HIV transmission at the point of contact. Taken together, PA and 2-BP binding to the CD4 cavity represents a platform for scaffold-based drug development.

The scaffold molecule PA has been shown to be associated with human disease. We therefore decided to test for genotoxic potential of PA and 2-BP scaffolds (Table 1, Fig. 1 and 2). In the bacterial reverse mutation assay (Ames test) neither PA or 2-BP tested up to the maximum concentration of 5000 μg/plate did not exceed two fold number of revert mutants as compared to positive control, indicating that the test articles are not mutagenic in bacteria (Table 1). Likewise, in the MLA, both molecules did not induce number of mutations necessary to be considered positive for mutagenesis in eukaryotic cells (Fig. 1). For the *in vivo* gene mutagenic potential, we tested scaffolds in the Rat Micronucleus Assay (Fig. 2). Neither PA or 2-BP significantly inhibited erythropoiesis or increased MPCE’s demonstrating that both molecules do not induce *in vivo* gene mutations in test animals. However, in the confirmatory study, 2-BP caused mortality at the highest recommended dose of 2000 mg/kg, and PA was not toxic. This difference may be explained in different metabolic pathways of the two test articles. PA is metabolized in mitochondria into acetyl CoA, a substrate of the TCA cycle, by fatty acid beta-oxidation [33]. However, 2-BP is non-metabolizable analog of PA and may contribute to cell toxicity by inhibiting mitochondrial oxidation of fatty acids, which would explain 2-BP mortality that was observed at highest 2000 mg/kg recommended dose, leading to testing at 1800 mg/kg dose in the definitive study. Meeting historical criteria validated all tests performed, and even at the highest compound concentrations in each assay, there were no positive genotoxic results and no concentration based changes in mutant frequency. Each test was also performed with metabolic activation indicating that the metabolites produced from hepatic metabolism of the compounds are also non-mutagenic. These results, along with the *in vivo* rat micronucleus assay demonstrate the non-mutagenicity of PA and 2-BP in complex living organisms.

Collectively, based on the results we conclude that PA and 2-BP model scaffolds are safe and validate further development of HIV entry inhibitors with similar structures. Further docking studies are being performed to better understand the SAR in order to create a more directed approach to lead discovery and optimization.
